# Unraveling Cell of Origin in Breast Cancer via Joint Profiling of Whole Genome and Transcriptome in Individual Cells

**DOI:** 10.1093/gpbjnl/qzag021

**Published:** 2026-03-03

**Authors:** Yueying He, Ruli Gao

**Affiliations:** Department of Biochemistry and Molecular Genetics, Northwestern University Feinberg School of Medicine, Chicago, IL 60611, USA; Center for Cancer Genomics, Robert H. Lurie Cancer Center, Northwestern University Feinberg School of Medicine, Chicago, IL 60611, USA; Department of Biochemistry and Molecular Genetics, Northwestern University Feinberg School of Medicine, Chicago, IL 60611, USA; Center for Cancer Genomics, Robert H. Lurie Cancer Center, Northwestern University Feinberg School of Medicine, Chicago, IL 60611, USA; The Driskill Graduate Program, Northwestern University Feinberg School of Medicine, Chicago, IL 60611, USA

## Background

Profiling both the whole genome and transcriptome from the same single cells poses a significant technical challenge in resolving the epithelial cell-of-origin in diverse breast cancer subtypes. Existing co-profiling technologies are often suboptimal due to the challenges in isolating high-quality DNA and RNA molecules from a cell and lack of the capacity for barcoding both molecular layers at scale [1–6]. Computational inference of DNA copy number alterations (CNAs) from single-cell RNA sequencing (scRNA-seq) data [7–9] paves the way to simultaneously measure genotype and phenotype in individual cells, which, however, is constrained by the levels of gene dosage effects, resulting in low genomic resolution and varied accuracy. To address these, Wang et al. [10] developed wellDR-seq, a Nanowell technology for direct sequencing of both CNAs and transcriptomes in individual cells. By applying wellDR-seq to 12 estrogen receptor‑positive (ER^+^) breast cancer patients, the authors decoded several critical questions regarding breast cancer progression, including the identification of ancestral cancer subclones and their epithelial lineages, normal epithelial and stromal cell states with somatic CNAs, and gene dosage effects of CNAs among subclones.

## A robust technology for co-profiling single-cell whole-genome CNAs and transcriptomes

The new wellDR-seq method starts with loading single-cell suspensions into a 5184-Nanowell chip, followed by automated image scanning and selection of desired Nanowells containing viable single cells, at a scale of 1200–2600 cells per run. Individual cells within each Nanowell are then chemically processed to release DNA and RNA molecules, in which a specific treatment is applied to remove chromatin from DNA molecules. Both DNA and RNA molecules are subject to double-round modality-specific barcoding and cell-specific barcoding using the 72 × 72 multiplexing strategy. The barcodes for RNA and DNA modalities are designed either with or without biotinylation to distinguish the two molecular layers in high-throughput next-generation sequencing. Of note, this separation strategy allows for direct measurement of single-cell DNA copy numbers, reducing both missed and spurious CNA events relative to the computational inference from RNA data [7–9]. Indeed, the authors showed that computational inferences [7–9] often failed to capture minor subclonal CNA events. Furthermore, they performed a series of benchmarking and cell-line spike-in wellDR-seq, which demonstrated its capacity for recovering cell populations at various frequencies, including 50%, 20%, 10%, 5%, and even 1%. Together, these data highlight the unique strength of directly co-measuring DNA and RNA using the experimental approaches developed for wellDR-seq, which allows for establishing more precise connections between genotypes and phenotypes in the same cells.

## Detecting luminal hormone-responsive cells as the potential cell-of-origin in ER^+^ breast cancer

To investigate the origin of cancer cells, single-cell RNA data were used to identify the cancerous and normal epithelial phenotypes, while matched DNA data of individual cells were used to track their evolutionary precursors. Applying wellDR-seq to an ER^+^/progesterone receptor‑positive (PR^+^) patient, the authors identified a cluster of luminal hormone-responsive (LumHR) cells as the potential ancestral population, because the only CNA event on Chr22 in this cluster was found in all cancer cells but not in other normal LumHR cells. Similarly, in a synchronous ductal carcinoma *in situ* (DCIS) and invasive ductal carcinoma (IDC) sample, they found a rare clone with CNAs on Chr16q as the ancestral population of a more expanded subclone. Similarly, the rare subclones displaying a high LumHR phenotype but not luminal secretory (LumSec) or basal-myoepithelial (MyoEpi) gene modules were revealed as ancestral cells in two other ER^+^/PR^+^ patients. Furthermore, the aneuploid cancer cells showed weaker activation of the LumHR phenotype compared to their ancestral normal cells. These data imply that lineage tracing based solely on gene expression programs may have varied reliability due to transcriptional plasticity, justifying the need for more stable genetic markers to delineate cell-to-cell evolutionary relationships in cancer. In addition, the authors identified multiple rare cell clusters harboring sporadic CNA events on autosomes in noncancerous epithelial cells, whereas the stromal cells, including fibroblasts, pericytes, and endothelial cells, uniquely gained sporadic CNA events on ChrX. Notably, all cells with sporadic CNAs showed low cellular frequencies in these patients, suggesting limited fitness advantages of these cells within the tumor microenvironment. In summary, while the aneuploid cancer cells are likely derived from a subset of LumHR cells, the noncancerous epithelial cells with sporadic CNAs are detected in both LumHR and LumSec lineages.

## Investigation of copy-number dosage effect at both individual gene and segmental levels

To further investigate the gene dosage effects of CNAs in breast cancer, the authors analyzed the correlations between CNA events and gene expression profiles in the most abundant subclones of aneuploid cancer cells from 11 ER^+^ samples. The integrative mapping results suggested that a large proportion (∼ 69%) of differentially expressed genes (DEGs) between subclones may be regulated by other mechanisms rather than copy-number dosage effects. Based on the correlation measurements between copy-number status and expression status of individual genes, they further classified DEGs between subclones into dosage-sensitive and dosage-insensitive events. Interestingly, this stratification approach revealed a consistent increase in the proportion of dosage-sensitive genes when the copy-number differences became larger, in which the dosage effects reached statistical significance. Among breast cancer-specific genes, they found that *PGR*, *AURKA*, and *RB1* were dosage-sensitive genes, whereas *PIK3CA*, *BRCA1*, and *TP53* did not show a significant correlation with their copy numbers. Normalizing gene expression levels to diploid copy-number status revealed a much higher dosage effect in dosage-sensitive genes (*r* = 0.62) compared to dosage-insensitive genes (*r* = 0.17). At the segmental level, their data exhibited a high correlation between copy-number status and gene expression, where 55.7% of the DNA segments had corresponding gene expression changes with the median gene expression difference (MGED) greater than 0.03. The authors also found that a large amount of variation in gene expression was not associated with copy-number status, implying other gene regulation mechanisms in breast cancer.

## Closing remarks

Wang et al. [10] report a high-resolution and high-throughput method (wellDR-seq) that can simultaneously measure whole-genome copy numbers and transcriptomes in thousands of individual cells. Benchmarking analyses demonstrate that wellDR-seq achieves robustness in direct profiling of both DNA copy numbers and gene expression in single cells, with high sensitivity in detecting rare cell populations. In the applications, wellDR-seq identifies the cell of origin in ER^+^ breast cancer patients by tracing the shared CNA events between the ancestral normal LumHR cells and the expanded aneuploid cancer cells. These critical findings are made possible by establishing the direct links between DNA and RNA profiles in individual cells with wellDR-seq. Two major limitations of this technology are noted: (1) single-cell DNA sequencing has low capacity for detecting genome-wide single nucleotide variants (SNVs) due to the limited coverage breadth and depth at the single-cell level, and (2) the scRNA-seq library does not harbor unique molecular identifiers (UMIs), requiring extra efforts to normalize experimental artifacts for better quantification of gene expression data. Aside from these limitations, wellDR-seq uniquely enables the direct measurement of DNA and RNA in each cell, allowing precise analysis of the relationships between CNAs and gene expression programs at both the clonal and single-cell levels.

## CRediT author statement

**Yueying He:** Writing – review & editing. **Ruli Gao:** Conceptualization, Writing – original draft. Both authors have read and approved the final manuscript.

## Competing interests

The authors have declared no competing interests.
